# Achievement Goals, Fear of Failure and Self-Handicapping in Young Elite Athletes with and without Chronic Pain

**DOI:** 10.3390/children8070591

**Published:** 2021-07-12

**Authors:** Bodile Molenaar, Charlotte Willems, Jeanine Verbunt, Mariëlle Goossens

**Affiliations:** 1 Department of Rehabilitation Medicine, Care and Public Health Research Institute, Maastricht University, Universiteitssingel 40, 6229 ER Maastricht, The Netherlands; b.molenaar@maastrichtuniversity.nl (B.M.); charlotte.willems88@gmail.com (C.W.); jeanine.verbunt@maastrichtuniversity.nl (J.V.); 2 Adelante Center of Expertise in Rehabilitation and Audiology, Zandbergsweg 111, 6432 CC Hoensbroek, The Netherlands; 3 Department of Clinical Psychological Sciences, Experimental Psychology, Maastricht University, Universiteitssingel 40, 6229 ER Maastricht, The Netherlands

**Keywords:** elite athletes, chronic pain, self-handicapping, goal orientation, fear of failure

## Abstract

Background: Pain is a common problem in elite athletes. This exploratory study compares goal orientations towards sport, fear of failure, self-handicapping and pain catastrophizing between active young elite athletes with and without chronic pain (CP) complaints (longer than three months). It examines the associations between chronic pain, fear of failure, goal orientations, self-handicapping and pain catastrophizing in young elite athletes. We explore how far goal orientation can be explained by these factors. Methods: Young elite athletes completed an online questionnaire. Data analysis: Independent samples *t*-test, correlational analyses and multivariate regression analyses. Results: Participants were 132 young elite athletes (mean 16 years); data for 126 were analyzed. A total of 47% reported current pain, of which 60% had CP. Adolescents with CP showed significantly more pain intensity, fear of failure, self-handicapping and mastery–avoidance goals than those without. Pain intensity was significantly related to fear of failure, self-handicapping, pain catastrophizing and mastery–avoidance. Self-handicapping and fear of failure contributed significantly to mastery–avoidance variance. Performance–avoidance and –approach goals were explained by fear of failure. Conclusion: CP was common, with sufferers showing more fear of failure and self-handicapping strategies, and being motivated to avoid performing worse (mastery–avoidance). Self-handicapping and fear of failure influenced mastery–avoidance orientation, and fear of failure explained part of performance–avoidance and –approach orientations. Longitudinal studies should explore the role of these factors in the trajectory of CP in these athletes.

## 1. Introduction

Chronic, non-specific pain is a common health problem among children and adolescents [1,2,3]. Although prevalence rates vary substantially across studies, median prevalence rates of chronic pain range from 11 to 38%, are higher in girls than in boys and tend to increase with age [1,2]. Among the pain conditions, chronic headache, musculoskeletal pain and abdominal pain are the most common in children and adolescents in the Netherlands [4].

The impact of chronic pain in adolescents is tremendous. It is often associated with emotional distress and disability [5,6]. Adolescents frequently report experiencing dysfunction in physical, social and academic aspects of life [6,7,8,9,10,11]. Thus, chronic pain leaves its marks in the life of the adolescent. The fear-avoidance model of chronic pain and the interpersonal fear-avoidance model have unraveled several contributing factors for pain-related disability, such as pain-related fear, pain catastrophizing and the interaction between adolescents and their family systems [12,13]. Interdisciplinary exposure in vivo has proved successful in decreasing pain-related fear and functional disability in this adolescent chronic pain population [14].

Clinical observation of adolescents with chronic pain in a rehabilitation setting has revealed an important subgroup, namely young former elite athletes. These youngsters are no longer able to play sports at elite level due to their pain and appear limited in daily life activities and functioning. Since their route to becoming chronic pain patients and their vulnerabilities may differ from those of other adolescents with chronic pain, it is important to identify specific disabling factors for this subgroup. Insight into these factors may provide more tools for treating this group of adolescents and may help determine whether specific elements should be added to treatment packages for disabling chronic musculoskeletal pain. 

In elite sport, injuries and pain symptoms appear not merely to be associated with physical distress, but also with psychological symptoms due to high training loads and the high amount of pressure to which young athletes are exposed [15,16]. Previous studies have shown that vulnerability to dropout and the performance determinants of elite athletes are related to factors such as goal orientation, fear of failure and self-handicapping [17,18,19,20,21,22,23,24,25,26,27,28,29,30,31]. These findings will be addressed below.

Athletes can have different goal orientations through which they evaluate and interpret their competence and success. According to the achievement goal theory of Elliot and Church [17], type of motivation and orientation towards a goal are related to success and failure in sports and may be important in explaining dropout in young athletes [18,19,20]. The achievement goal theory comprises a 2 × 2 framework comprising two dimensions: mastery versus performance and approach versus avoidance. In the mastery construct, competence is defined in terms of absolute and intrapersonal competence; in the performance construct, competence is defined in normative terms and by competence relative to others. Crossing the dimensions of the achievement goal framework, four types of achievement goals can be differentiated: (1) mastery–approach, (2) mastery–avoidance, (3) performance–approach and (4) performance–avoidance [19]. From the different goals, the mastery–approach goals, which are centered on attaining task- or self-referenced competence, are considered the most beneficial in achievement settings. Successful elite athletes are highly driven by personal (mastery) goals, and have an intrinsic and self-determined motivation and skills-enhancing focus [17,18]. These athletes focus on improving their own skills and techniques, enjoyment and personal achievements. Mastery goals are task-oriented and beneficial to perseverance in high-performance-level sports [21]. Mastery–avoidance goals represent the motivation to avoid absolute or intrapersonal incompetence, such as striving to avoid doing worse than one has done previously. Performance goals are ego-oriented: performance–approach goals focus on attaining normative competence, such as striving to beat the opponent, or being special or popular, whereas performance–avoidance goals focus on avoiding negative outcomes, such as trying to avoid doing worse than others [20]. 

Furthermore, in addition to the need for achievement, fear of failure energizes achievement behavior and predisposes individuals to adopt particular types of achievement goals [22]. Fear of failure is conceptualized as the motive to avoid failure in evaluative situations, associated with shame and with the tendency to appraise threat, and is already present at a young age [23]. In elite sports, especially with young elite athletes who are more sensitive to failure and criticism, fear of failure is highly prevalent [24]. A possible consequence of fear of failure is that the athlete may modify his or her motivation and achievement goals, in order to reduce this fear. Motivation may become more ego oriented and avoidance related [22,25,26]. More specifically, fear of failure is related to performance–avoidance and performance–approach achievement goals [26]. Since sport represents a significant achievement and evaluation domain, concerns about performance failure are a major source of perceived psychological stress [27]. A strategy aiming at managing self-image, associated with fear of failure, is self-handicapping. 

Self-handicapping is a proactive strategy that involves constructing or reporting impediments before entering a performance situation, in order to protect one’s self-esteem in the event of failure or to enhance one’s self-esteem in the event of success [28,29]. Anticipating possible failure, athletes may give excuses or may identify possible obstacles. These can be expressed in task avoidance, withholding effort, lack of practice, reporting illness or other physical symptoms and the choice of other performance-limiting circumstances [30,31]. Although self-handicapping may protect one’s sense of self-worth in the short term, in the long term this strategy may lead to the feeling of failure that one was trying to avoid. This feeling of failure would then confirm their doubts about their capacities, resulting in a downward spiral that might promote the use of self-handicapping strategies and avoidance. A reduction in training due to, for example, a minor sporting injury could be used as a self-handicapping justification. This can result in poorer condition and, when training is resumed, muscle pain. This pain could reinforce the athlete’s worries about (the chance of) re-injury, disrupting further training and encouraging negative cognitions about the pain.

In our clinical observations, the chronic pain of former elite athletes may be a product of goal orientation, fear of failure and self-handicapping, expressed in complaints of pain. In addition, pain catastrophizing may contribute to the process of chronification of the pain and finally make it impossible to continue sport [12,13]. 

This explorative study had three aims. The first was to compare goal orientations towards sport, fear of failure, self-handicapping and pain catastrophizing between active young elite athletes with and without chronic pain. The second aim was to explore associations between self-reported pain intensity, different types of goal orientations, fear of failure, self-handicapping and pain catastrophizing. The third aim was to study the extent to which goal orientation strategies can be explained by pain intensity, fear of failure, self-handicapping and pain catastrophizing. 

We hypothesized that athletes with chronic pain would show increased levels of self-handicapping, fear of failure, pain catastrophizing and the less successful achievement goals (performance–approach, performance–avoidance and mastery–avoidance) (H1). Furthermore, we expected to find associations between pain intensity, pain catastrophizing, self-handicapping, fear of failure and the less successful achievement goals (H2). Finally, we hypothesized that levels of fear of failure, self-handicapping, pain intensity and pain catastrophizing would account for a substantial part of the less beneficial goal orientations (H3). 

The results of this study could be important in the support of young elite athletes and the prevention of dropout from elite sports. The outcomes could also help us elaborate on the pathway to chronic pain in former elite athletes.

## 2. Methods

### 2.1. Participants and Procedure

Participants in this cross-sectional study were young elite athletes selected at regional, national or international sports levels. They were recruited from professional sports clubs in Flanders, Belgium. Participants were aged between 12 and 21 years. To enable contact with young elite athletes, support was given by Sport Vlaanderen, the first port of call for professional sports clubs and elite athletes in Flanders and supported by the Flemish government. Potential participants were invited by their own sports clubs, the invitations including an information letter about the content of the study, directed to the young elite athlete and to his or her parents. After informed consent, participants were asked to complete an online questionnaire, taking some 20 min. The study was approved by the Ethics Review Committee Psychology and Neuroscience (ERCPN). 

### 2.2. Measures

The online questionnaire (distributed with Qualtrics) used in this study comprised a battery of assessment instruments for measuring pain intensity, fear of failure, goal orientation, self-handicapping and pain catastrophizing. Questions about socio-demographic and medical variables, as well as sports characteristics, were included. 

### 2.3. Socio-Demographics and Sports

The socio-demographic variables measured in this study were age and gender. 

Sports variables included in the questionnaire were type of sport, starting age, competition frequency and performance satisfaction. Type of sport was divided into four categories: (1) individually measurable sports (e.g., athletics, cycling, swimming, sailing, ice-skating, skiing); (2) team sports (e.g., soccer, handball, volleyball, basketball, hockey, ice hockey, baseball); (3) martial arts and jury sports (e.g., judo, karate, taekwondo, boxing, gymnastics); and (4) individual ball sports (e.g., tennis, table tennis, golf, squash, badminton). Competition frequency was measured as number of competition days over the past year. 

### 2.4. Current Pain Complaints and Pain Duration

Current pain intensity was measured with the Visual Analogue Scale (VAS), ranging from 0 (“*no pain at all*”) to 10 (“*maximum/unbearable pain*”) [32] in response to the question: “*How much pain do you have at this moment?*”. Athletes were also asked how long ago the current pain complaints had started. Athletes with a pain onset longer than three months ago were classified as having chronic pain. Furthermore, athletes were asked whether they believed their pain was caused by their sports activities and, further, whether they had a close relative with chronic pain. 

### 2.5. Fear of Failure

The Performance Failure Appraisal Inventory [33] (PFAI) was administered to measure fear of failure. This inventory comprises 25 items measuring beliefs that failure is followed by five aversive consequences: (a) experiencing shame and embarrassment; (b) devaluing one’s self-estimation; (c) having an uncertain future; (d) losing the interest of important others; and (e) upsetting important others. Sample items included (a) “*When I am not succeeding, I am less valuable than when I succeed*”, (b) “*When I am failing, I blame my lack of talent*”, (c) “*When I am failing, my future seems uncertain*”, (d) “*When I am not succeeding, people are less interested in me*” and (e) “*When I am failing, I expect to be criticized by important others*”. Responses were made on a five-point scale ranging from “*do not believe at all*” (−2) to “*believe 100% of the time*” (+2). For analysis, an average score over the PFAI subscales was calculated as the total score for fear of failure, ranging from −2 to +2. Higher scores indicate higher fear of failure. In earlier studies, the PFAI demonstrated good construct validity and reliable internal consistency [22,34]. In the present study, the internal consistency of the PFAI was excellent (α = 0.93).

### 2.6. Sports Goal Orientation

Sports goal orientation was measured through the Dutch Goal Orientation Questionnaire (Nederlandstalige Doeloriëntatie Vragenlijst [35]; NDV), a translation of Elliot and McGregor’s (2001) Achievement Goals Questionnaire, based on the 2 × 2 achievement goal framework. This 12-item measure provides scores for mastery–approach (M–Ap), mastery–avoidance (M–Av), performance–approach (P–Ap) and performance–avoidance (P–Av) achievement goals. In line with the study of Conroy, Elliot and Hofer [26], in each item, “study” was replaced by “sport” (Achievement Goals Questionnaire-Sport; AGQ-S). Sample items included “*It is important for to me to learn as much as possible in my sport*” (M–Ap), “*I worry that I may not perform as well as I possibly can*” (M–Av), “*It is important to me to do well compared to others*” (P–Ap) and “*My goal is to avoid performing worse than others*” (P–Av). Responses were made on a 7-point scale ranging from “*not at all true for me*” (1) to “*very true for me*” (7). Each of the four types of goal orientation was measured through 3 items. Average scores of subscale items were calculated as a subscale score, ranging from 1 to 7. Higher scores indicate a higher degree of the goal orientation type concerned. The AGQ-S has been used in several sports-related studies and has demonstrated good psychometric properties [22,26]. In the present study, internal consistency of the NDV subscales of M–Ap (α = 0.74), M–Av (α = 0.83) and P–Ap (α = 0.79) ranged from acceptable to good. Internal consistency of the P–Av subscale was questionable (α = 0.60).

### 2.7. Self-Handicapping

Self-handicapping was assessed through the Self-Handicapping Scale [36] (SHS). The translation of the Dutch SHS was checked through the process of back-translation. The SHS is a 25-item questionnaire, designed to measure individual differences in self-handicapping, and focuses mainly on the self-protective aspect of self-handicapping. The items of the scale measure the respondent’s tendency to use such self-handicapping behaviors as lack of effort (“*I would do a lot better if I tried harder*”), illness (“*I suppose I feel ‘under the weather’ more often than most people*”), or procrastination (“*I tend to put things off to the last minute*”). Responses were made on a 6-point scale from “*I totally disagree*” (1) to “*I totally agree*” (6). Average scale scores, ranging from 1 to 6, were used for analysis. Higher scores indicate the presence of greater levels of self-handicapping. In the present study, the internal consistency of the SHS was questionable (α = 0.65).

### 2.8. Pain Catastrophizing

Children’s catastrophic thinking about pain (i.e., an exaggerated negative orientation towards actual or anticipated pain experiences) was measured with the Pain Catastrophizing Scale for Children [37] (PCS-C). This comprises 13 items describing different thoughts and feelings that children may experience when they are in pain. Three subscales are distinguished: (a) rumination (“*When I am in pain, I keep thinking about how much it hurts*”), (b) magnification (“*When I am in pain, I keep thinking of other painful events*”), and (c) helplessness (“*When I am in pain, I feel I can’t go on like this much longer*”). Items were scored on a five-point scale (0–4) and item scores were averaged into a general score for pain catastrophizing, ranging from 0 to 4. The Dutch PCS-C has been shown to be both reliable and valid for children [37]. In the present study, internal consistency of the PCS-C was excellent (α = 0.92).

### 2.9. Statistical Analyses

This was an observational study with a cross-sectional design. First, the data were checked on their distributions. Apart from mastery–approach, all variables were normally distributed with skewness and kurtosis between −1.00 and 1.00. To test differences between the scores of elite athletes with chronic pain compared to those without chronic pain (H1), t-test analyses were used. Then, Pearson correlations were calculated to examine the relationship between pain intensity, fear of failure, self-handicapping, pain catastrophizing and goal orientation subtypes (H2). For mastery–approach, Spearman correlations were used. Finally, to study the extent to which the different types of goal orientation strategies can be explained by pain intensity, fear of failure, self-handicapping and pain catastrophizing (H3), we used multivariate regression analyses. Dependent variables were mastery–approach, mastery–avoidance, performance–approach and performance–avoidance goal orientations. Pain intensity, self-handicapping, fear of failure and pain catastrophizing were entered as independent variables. Collinearity control included checking variance inflation factors, which had to be below 10. IBM SPSS Statistics version 25 was used for statistical analyses.

## 3. Results

### 3.1. Description of the Study Population

A total of 132 young elite athletes, 71 boys and 61 girls, participated in this study. Mean age was 16.3 years (SD (Standard Deviation) = 2.7, range 12–21). 

Demographics, sport- and pain-related variables are displayed in Table 1. Of the participants, 57.6% started their sport at a young age, 4 to 7 years, with the greatest percentage (18.2%) at 4. Most practiced team sports (47.0%), followed by individually measurable sports (32.6%), martial arts and jury sports (10.6%) and individual ball sports (9.8%). The majority were active in national or international competitions (49.2 and 43.9% respectively), with a small number only participating in regional competitions (3.8%). Eighty-three percent of the elite athletes were satisfied with their sporting achievements. 

Current pain complaints in the musculoskeletal area were reported by 46.8% of the athletes, with a quarter of these reporting a pain duration of less than one month and almost 60% reporting chronic pain (lasting longer than three months). Of the athletes reporting pain, 75% were convinced and 19% thought it likely that their complaints resulted from their sporting activities. Fifty-seven percent of all athletes had a close relative with chronic pain. Mean pain intensity for all athletes was 2.01 (SD 2.05) which could be described as mild [38]. 

Table 2 shows the means and standard deviations for pain intensity, fear of failure, self-handicapping, pain catastrophizing and goal orientation subtypes of the total group of athletes and of athletes with and without chronic pain. Athletes reported very high levels of mastery–approach goals, moderate levels of performance–approach and performance–avoidance goals and moderate low levels of mastery–avoidance goals [39]. Mean scores for mastery–approach, performance–approach and performance–avoidance goal orientations were comparable to the mean scores of university athletes in Conroy and Elliot’s study [22]. However, the mean score for mastery–avoidance goal orientation in the sample was considerably lower than in university athletes [22,39]. Scores for fear of failure and self-handicapping were moderate and similar to those for undergraduate student populations performing sports [25,40].

Despite their pain complaints, the elite athletes with chronic pain still participated in training and competition. However, while athletes with and without chronic pain participated equally in competitions, those with chronic pain spent fewer hours in training. 

### 3.2. Comparison of Fear of Failure, Self-Handicapping, Pain Catastrophizing and Goal Orientations between Athletes with and without Chronic Pain

In line with H1, *t*-test analyses demonstrated that athletes with chronic pain reported higher levels of pain intensity and pain catastrophizing thoughts and scored more highly for fear of failure and the use of self-handicapping strategies than those without (see Table 2). Athletes with chronic pain were also significantly more mastery–avoidant than those without. This means that they strove to avoid performing worse than they had done before (probably without pain). Contrary to H1, performance–approach and performance–avoidance goals were not significantly different between athletes with and without chronic pain. 

### 3.3. Relationships between Pain Intensity, Fear of Failure, Self-Handicapping, Pain Catastrophizing and Goal Orientations

Pearson’s correlations between pain intensity, fear of failure, self-handicapping, pain catastrophizing, mastery–approach, mastery–avoidance, performance–approach and performance–avoidance validated H2. The higher the pain intensity of the athletes, the more the negative thoughts about pain, the more they feared failure and the more they used self-handicapping strategies (see Table 3). There also appeared to be a strong relationship between pain intensity and mastery–avoidance goal orientation, indicating that adolescents with more pain strove to not perform worse than before. In addition, fear of failure in athletes appeared to be significantly related to self-handicapping and mastery–avoidance, performance avoidance and performance–approach goal orientations. This indicates that the goals of athletes with higher fear of failure were more directed towards avoiding worse performance than before. Besides this, they also avoided performing worse than their opponents and strove to perform better than others.

### 3.4. Prediction of Goal Orientations 

To test possible predictors of mastery–approach, mastery–avoidance, performance–approach and performance–avoidance goals, we conducted multivariate regression analyses. The results were partly consistent with H3 (see Table 4). Mastery–approach goals were negatively predicted by self-handicapping only (β = −0.24; *p* = 0.03), though the total model was not significant. Mastery–avoidance goals were positively predicted by self-handicapping strategies (β = 0.45; *p* = < 0.01) and fear of failure (β = 0.23; *p* = < 0.01). The total model explained 44% of the variance in mastery–avoidance goals. Performance–approach and performance–avoidance goals were positively predicted by fear of failure only (β = 0.34; *p* = < 0.01); (β = 0.37; *p* = < 0.01) and accounted for 7 and 9% of the variance in these goals, respectively. Pain intensity as well as pain catastrophizing did not explain goal orientations in the adolescent athletes. Introduction of gender as an independent variable in the regression model, as suggested by Morris and Kavussanu [39], showed that there was no significant additional effect on any of the four goal orientations (*p* > 0.05).

## 4. Discussion

Chronic pain is a major and growing health problem in adolescents [1,4]. Pain is also a common problem in elite athletes, mainly due to sports injuries [41,42]. Due to psychosocial factors, the pain may develop or persist independently of the injury and lead to dropout from sports and daily life activities. The first aim of the present study was to compare goal orientations towards sport, fear of failure, self-handicapping and pain catastrophizing between active young elite athletes with and without chronic pain. The second aim was to explore the relationships between pain intensity, fear of failure, goal orientation subtypes, self-handicapping and pain catastrophizing. The third aim of this study was to explore the extent to which goal orientation strategies could be explained by pain intensity, fear of failure, self-handicapping and pain catastrophizing. 

The findings of this study provide important new insights by showing that athletes with chronic pain report higher levels of pain, fear of failure, self-handicapping behavior and mastery–avoidant goal orientation strategies than those without. The study revealed that pain intensity was positively associated with pain catastrophizing, fear of failure, self-handicapping strategies and mastery–avoidance goals. Finally, a substantial part of these mastery–avoidance goals were explained by self-handicapping and fear of failure. In addition, fear of failure appeared to be a significant predictor of performance–approach and performance–avoidance goal orientations.

This group of young elite athletes was active in different types of sports and performed mainly at national or international levels. They had moderate levels of fear of failure and self-handicapping, comparable to university athletes [25,40]. In line with previous studies, all four types of goal orientations were common [22,39], which is positive for athletes performing at high levels. Having a high achievement goals profile (i.e., high levels of all four goal orientations) ensured more adaptive motivational patterns [43]. As expected in this group of athletes performing at the highest level, mastery–approach goal orientation was the most strongly present. Goals in this achievement orientation are centered on one’s own skills and linked to positive processes and outcomes, found to be most beneficial in achievement settings [22,44]. In addition, mastery–approach goal orientation in young, competitive athletes was also linked to continuation of sports in the long term [45]. 

Mild musculoskeletal pain complaints were reported by almost half of the group of elite athletes, who were convinced that these pain complaints were sports related. Pain intensity was positively associated with pain catastrophizing, fear of failure, self-handicapping strategies and mastery–avoidance goals orientation. Since sports injuries are frequently accompanied by pain in elite athletes, the percentage of athletes reporting pain is not surprising [42,46]. In training and competition, pain may result from straining, from physical contact with other athletes or from injuries occurring during sporting activities. It is, however, alarming that 50% of the athletes reported having these complaints longer than three months, exceeding the normal healing time for most common muscle strains [47]. During this period, the pain may have interrupted training and interfered with sports- and competition-related activities. Studies in the field of chronic pain have shown that long-lasting pain complaints can lead to limitations in daily functioning and lower quality of life, also increasing the risk of pain in adulthood [48,49]. Under the influence of pain-related fear and negative thoughts about pain, fearful people anticipate (re)injury to their body, and react by avoiding certain movements [12,48]. Furthermore, in injured athletes, psychosocial factors, such as symptoms of depression, fear and pain catastrophizing, were associated with prolonged recovery time and higher risk of re-injury [46,50]. 

In our study, pain catastrophizing did not differ between athletes with and without chronic pain complaints. Those with chronic pain, however, scored significantly higher for fear of failure, the use of self-handicapping strategies and mastery–avoidance goals. Looking at the training and competition frequency of the athletes, we saw that, despite chronic pain, athletes continued to train and to participate in competitions. However, the number of training hours was noticeably lower for athletes with chronic pain than for those without. This was in contrast with competition frequency, which hardly differed between the two groups. In the group with chronic pain, this may be used as a reason to train less, providing a ready-made excuse should failure or poor performance occur. Mastery–avoidance was very strongly related to self-handicapping, indicating that the more the athlete tries to avoid performing worse than before, the more they use self-handicapping strategies. Lack of practice is a commonly used behavioral self-handicapping strategy for poorer performance [30,51,52]. In addition, as pain complaints are subjective and unobservable to others, in the long run, these can serve as a self-reported self-handicapping strategy. In our athlete population, pain intensity was positively associated with self-handicapping, as well as with pain catastrophizing, indicating that a higher level of pain is associated with more self-handicapping behavior and more pain catastrophizing. This finding is in line with the study of Uysal and Lu [53], showing a positive association between self-handicapping, catastrophizing and self-reported pain. The use of self-handicapping strategies has a negative effect on performance [52,54]. Although we have no insight into the sports performance of our athletes, we can assume that fewer hours of training will have led to poorer performances. Surprisingly, nearly all adolescents reported being satisfied with their sporting performances. However, this was measured dichotomously (“yes/no”) so the opportunity for reporting variation in performance satisfaction was not provided. The option “yes” would presumably have been chosen by those with at least some satisfaction with their performance; few would have reported no satisfaction at all (and perhaps some of these would have quit their sport already).

In addition, since fear of failure is conceptualized as the motive to avoid failure in negative situations, it was not surprising that the use of self-handicapping strategies was positively associated with fear of failure in the elite athletes, consistent with previous findings [55,56]. Persistent pain complaints, fear of failure and the use of self-handicapping strategies could explain the prevalence of mastery–avoidant goal orientation strategies in athletes with chronic pain, compared to those without. Athletes with chronic pain tried to avoid performing worse than previously and used more self-handicapping strategies, such as the lack of practice, as ready-made reasons for their possible failure or lack of success. Focusing on mastery goals implies focusing on one’s progress and is typically found less threatening. However, a recent study of Coudevylle and colleagues [57] in college students showed that focusing on mastery goals does not remove the threat to one’s self-image, as there is still a fear of regressing. For young elite athlete with chronic pain, focusing on progress may also be highly threatening, since chances are high that they will perform worse than previously, as a consequence of their pain.

Through regression analyses, we explored self-reported pain intensity, self-handicapping, fear of failure and pain catastrophizing as predictors of the four types of goal orientation. In accordance with our hypotheses, the models were significant in predicting mastery–avoidance, performance–approach and performance–avoidance. The model’s contribution to mastery–avoidance goals was considerable. These goals were positively predicted by self-handicapping and fear of failure, indicating that athletes who use more self-handicapping strategies and have higher levels of fear of failure tend to try to avoid performing worse than they did previously [19]. Fear of failure also significantly contributed to performance–avoidance goals. Since fear of failure is conceptualized as the motive to avoid failure in evaluative situations, an association between fear of failure and both types of avoidance goal orientation was expected [22]. It predisposes athletes to adopt particular types of achievement goals. Fear of failure also appeared to be the only significant contributor in the model predicting both performance goal orientations. Relations between fear of failure and ego-oriented goals were in line with earlier findings [22,26].

Some findings did not correspond with our expectations. First, both pain catastrophizing and pain intensity did not predict a substantial part of our four goal orientations, even though there was a significant association between pain catastrophizing and intensity and mastery–avoidance goal orientation. For mastery–avoidance, the impacts of self-handicapping and fear of failure appeared to be stronger, making the contributions of pain intensity and catastrophizing inconsiderable. Second, contradicting previous findings [52,58], self-handicapping was not found to be a predictor of performance–avoidance goal orientation. We do not have an explanation for this finding, except for the questionable internal consistency of the NDV subscale assessing performance–avoidance goal orientation. Nevertheless, the internal consistency of the P–Av subscale was comparable with that in previous Dutch samples [35,59]. In addition, the internal consistency of the Self-Handicapping Scale (SHS) was questionable. This was not surprising, since the Cronbach’s alpha of the SHS is found to be generally low in studies conducted on athletes [60].

Several limitations of our study should be mentioned. First, the study was cross-sectional, which limits the interpretation of our results because of a lack of information about causality and the resulting uncertainty about the direction of the associations found. A longitudinal study in elite athletes would be more useful to assess causal relations between the persistence of pain and factors contributing to achievement goal orientations. Second, there is a chance that the prevalence of pain complaints in our group of young elite athletes has been overestimated. Adolescent athletes were invited to participate in a study into goal orientation, pain complaints and several psychological factors. It is possible that adolescents with pain complaints were triggered to participate in this study, while athletes without pain complaints were not. Nevertheless, the results of this study remain important because of the large number of sporting injuries among elite athletes and the need to prevent fear of failure and dropout in this group. Finally, it needs to be mentioned that this was an exploratory study in which only a limited set of variables was examined. In future studies, the influence of environmental factors, especially the roles of coaches and parents, should also be addressed.

Notwithstanding the limitations, this study is the first to our knowledge to examine the presence of and the association between (chronic) pain and goal orientation strategies, fear of failure, self-handicapping and pain catastrophizing in active young elite athletes. The study resulted in several interesting findings. Athletes with chronic pain, compared to those without, seemed to show more fear of failure and self-handicapping strategies, and had a self-determined motivation to avoid performing worse than previously. Self-handicapping and fear of failure also appeared to contribute to a substantial part of the variance of these mastery–avoidance goals in athletes both with and without chronic pain. Further longitudinal studies are required to examine the potential roles of fear of failure, self-handicapping and goal orientations in the trajectory of long-lasting pain complaints in young elite athletes. There is also a need to expand the exploration of environmental factors in this process. Family and coaches play an important role in the development of athletes’ goals, and of their coping strategies towards and maintenance of health issues, such as injury and pain [13,39,61]. In our study, 57% of athletes with current pain complaints appeared to have a close relative with chronic pain. Although the findings of the study are exploratory, it stresses the importance of a balanced set of goal orientation strategies in the support of young elite athletes. In line with the findings of Coudevylle and colleagues [57], it would be worthwhile to additionally consider self-determined goals in athletes with pain complaints, as these goals are more focused on needs and less on progress. As a consequence, these less threatening self-determined goals could decrease self-handicapping strategies and fear of failure [57]. Our study also emphasizes the importance of detecting fear of failure and self-handicapping strategies in young athletes, as these factors can lead to changes in behavior and goal orientations. The young elite athletes in our study were still actively performing at a high level; however, without addressing fear of failure, self-handicapping and alterations in behavior and goal orientations, pain complaints may persist and may finally result in dropout from sports, as well as negatively affecting other important life activities.

## Figures and Tables

**Table 1:** Demographics, sport- and pain-related variables for participants with and without chronic pain.

	All Adolescents (*n* = 132)	Adolescents with Chronic Pain Complaints (*n* = 35)	Adolescents without Chronic Pain Complaints (*n* = 91)
Age (years: mean ± SD)	16.3 ± 2.7 (range 12–21)	17.37 ± 2.5 (range 12–21)	15.8 ± 2.6 (range 12–21)
Gender (% male)	53.8%	48.6%	56.0%
Education level			
Primary education/low Middle High Unknown	11 (8.3%) 15 (11.4%) 99 (75.0%) 7 (5.3%)	- 5 (14.3%) 34 (82.9%) 1 (2.9%)	11 (12.1%) 10 (11.0%) 69 (75.8%) 1 (1.1%)
Starting age			
4 to 7 years 8 to 11 years >12 years	76 (57.6%) 37 (28.0%) 19 (14.4%)	18 (51.4%) 9 (25.7%) 8 (22.9%)	57 (62.6%) 24 (26.4%) 10 (11.0%)
Type of sport			
Team sports Individually measurable sports Martial arts or jury sports Individual ball sports	62 (47.0%) 43 (32.6%) 14 (10.6%) 13 (9.8%)	21 (60.0%) 10 (28.6%) 2 (5.7%) 2 (5.7%)	40 (44.0%) 32 (35.2%) 10 (11.0%) 9 (9.9%)
Performance level			
Regional competition National competition International competition Unknown	5 (3.8%) 65 (49.2%) 58 (43.9%) 4 (3.1%)	2 (5.7%) 14 (40.0%) 19 (54.3%) -	3 (3.3%) 51 (56.0%) 37 (40.7%) -
Training frequency (hours per week)			
<6 h 7–10 h 11–14 h 15–18 h >18 h Unknown	11 (8.3%) 42 (31.8%) 17 (12.9%) 13 (9.8%) 43 (32.6%) 6 (4.6%)	6 (17.1%) 10 (28.6%) 5 (14.3%) 2 (5.7%) 10 (28.6%) 2 (5.8%)	5 (5.5%) 31 (34.1%) 12 (13.2%) 10 (11.0%) 33 (36.3%) -
Competition frequency (days in the past year)			
<20 days 20–40 days 40–60 days 60–80 days 80–100 days Unknown	44 (33.3%) 48 (36.4%) 28 (21.2%) 4 (3.0%) 4 (3.0%) 4 (3.0%)	- 12 (34.3%) 14 (40.0%) 5 (14.3%) 1 (2.9%) 3 (8.6%)	- 32 (35.2%) 32 (35.2%) 23 (25.3%) 3 (3.3%) 1 (1.1%)
Relative(s) with pain complaints (% yes)	57.5% *	77.1%	49.5%

* Valid percent. SD = Standard Deviation

**Table 2:** Mean (SD) and *t*-test statistics for pain intensity, fear of failure, self-handicapping, pain catastrophizing and goal orientation subtypes for athletes with and without chronic pain.

	Total	Athletes with Chronic Pain Complaints	Athletes without Chronic Pain Complaints	*t*-Value	*p*-Value
Pain intensity (0/10)	2.01 (2.05)	3.33 (1.97)	1.50 (1.85)	4.53	<0.01 *
Fear of failure (−2/+2)	−0.60 (0.75)	−0.36 (0.71)	−0.69 (0.75)	2.12	0.04 *
Self-handicapping (1/6)	2.99 (0.45)	3.22 (0.34)	2.90 (0.46)	3.53	<0.01 *
Pain catastrophizing (0/4)	0.90 (0.74)	1.08 (0.59)	0.83 (0.78)	1.59	0.12
Mastery–approach goals (1/7)	6.31 (0.79)	6.31 (0.64)	6.30 (0.85)	0.05	0.96
Mastery–avoidance goals (1/7)	3.32 (1.58)	4.07 (1.43)	3.03 (1.55)	3.17	<0.01 *
Performance–approach goals (1/7)	4.96 (1.38)	5.11 (1.35)	4.90 (1.39)	0.71	0.48
Performance–avoidance goals (1/7)	4.46 (1.33)	4.47 (1.34)	4.46 (1.34)	−0.33	0.97

Note. Chronic pain = pain lasting longer than three months. * *p* < 0.05.

**Table 3:** Correlations between pain intensity, fear of failure, self-handicapping, pain catastrophizing and goal orientations.

	1	2	3	4	5	6	7	8
1. Pain intensity	---							
2. Fear of failure	0.21 *	---						
3. Self-handicapping	0.29 **	0.40 **	---					
4. Pain catastrophizing	0.38 **	0.18	0.22 *	---				
5. Mastery–approach	0.04	0.03	−0.22 *^#^	−0.01	---			
6. Mastery–avoidance	0.32 **	0.44 **	0.61 **	0.31 **	0.05	---		
7. Performance–approach	0.08	0.31 **	0.04	0.03	0.19	0.20 *	---	
8. Performance–avoidance	0.04	0.34 **	0.09	0.09	0.22 *^#^	0.31 **	0.37 **	---

Note. * *p* < 0.05. ** *p* < 0.01. ^#^ Spearman correlation.

**Table 4:** Multivariate regression analyses in young elite athletes with and without chronic pain predicting goal orientations.

Dependent Variable	Independent Variable	Adjusted R^2^	Standardized β	*p*-Value
Mastery–approach goals	Pain intensity Self-handicapping Fear of failure Pain catastrophizing	0.01	0.10 −0.24 * 0.11 0.04	0.25
Mastery–avoidance goals	Pain intensity Self-handicapping Fear of failure Pain catastrophizing	0.44 *	0.11 0.45 * 0.23 * 0.14	<0.01 *
Performance–approach goals	Pain intensity Self-handicapping Fear of failure Pain catastrophizing	0.07 *	0.08 −0.12 0.34 * 0.04	0.02 *
Performance–avoidance goals	Pain intensity Self-handicapping Fear of failure Pain catastrophizing	0.09 *	−0.05 −0.05 0.37 * −0.06	0.01 *

* *p* < 0.05.
